# The Matrikine Acetylated Proline-Glycine-Proline Couples Vascular Inflammation and Acute Cardiac Rejection

**DOI:** 10.1038/s41598-017-07610-0

**Published:** 2017-08-08

**Authors:** Gregory A. Payne, Jindong Li, Xin Xu, Patricia Jackson, Hongwei Qin, David M. Pollock, J. Michael Wells, Suzanne Oparil, Massoud Leesar, Rakesh P. Patel, J. Edwin Blalock, Amit Gaggar

**Affiliations:** 1 Division of Cardiovascular Disease, Department of Medicine, University of Alabama at Birmingham, Birmingham, AL USA; 2 Division of Pulmonary, Allergy & Critical Care Medicine, Department of Medicine, University of Alabama at Birmingham, Birmingham, AL USA; 3 Vascular Biology and Hypertension Program, University of Alabama at Birmingham, Birmingham, AL USA; 4 Program in Protease and Matrix Biology, University of Alabama at Birmingham, Birmingham, AL USA; 5 Lung Health Center, University of Alabama at Birmingham, Birmingham, AL USA; 6 Medical Service at Birmingham VA Medical Center, Birmingham, AL USA; 7 Department of Cell, Developmental, and Integrative Biology, University of Alabama at Birmingham, Birmingham, AL USA; 8 Department of Pathology and Center for Free Radical Biology, University of Alabama at Birmingham, Birmingham, AL USA; 9 Cardio-Renal Physiology and Medicine, Division of Nephrology, Department of Medicine, University of Alabama at Birmingham, Birmingham, AL USA

## Abstract

The extracellular matrix (ECM) is a dynamic, bioactive structure critical to organ development, structure and function. Excessive remodeling of the ECM is a hallmark of a variety of inflammatory conditions including vascular disease. Endothelin-1 (ET1) synthesis is understood to promote cardiovascular diseases including acute cardiac transplant rejection; however, the contribution of ECM-derived chemokines (matrikines) to vascular inflammation remains poorly understood. Herein we report that the matrikine acetylated Pro-Gly-Pro (PGP) stimulates vascular inflammation through activation of endothelial CXC Chemokine Receptor 2 (CXCR2) and production of endothelin-1 both *in vitro* and *in vivo*. As a proof of hypothesis, we demonstrate that coronary PGP levels associate with both circulating endothelin-1 and acute rejection in cardiac transplant patients (sensitivity of 100% and specificity of 86%). These findings establish PGP as a novel mediator in cardiovascular disease, and implicate bioactive matrix fragments as underappreciated agents potentially active in numerous conditions propagated by progressive vascular inflammation.

## Introduction

The extracellular matrix (ECM) is a well-accepted regulator of organ structure and function. However, ECM is increasingly understood to be a dynamic structure^1^, capable of modulating both local and systemic inflammation^2^. Importantly, dysregulation of the ECM has been implicated in the pathogenesis of numerous vascular-associated diseases including cancer^3^, heart failure^4^, coronary artery disease^5^ and stroke^6^. In particular, cardiac allograft vasculopathy and associated rejection represent a prototypical disease driven by disruption of immune, vascular and ECM regulation^7^. Together, these pro-inflammatory cascades lead to progressive cardiac graft dysfunction with limited therapeutic targets^8^.

ECM fragmentation and matrix metalloprotease (MMP) activity have been linked with arterial disease^9^, various cardiomyopathies^10^ and transplant associated complications^11^. Acetylated Proline-Glycine-Proline (PGP) is a novel tri-peptide, pro-inflammatory matrix-derived chemokine (matrikine) that was first characterized as a neutrophil chemoattractant^12^. PGP generated by MMP-8, -9 and prolyl endopeptidase mediated degradation of collagen can activate cell signaling via CXC-chemokine receptor 2 (CXCR2). PGP has recently been identified as a critical regulator of paracellular endothelial permeability in Acute Respiratory Distress Syndrome (ARDS)^13^ and promoter of neovascularization through human endothelial progenitor cell recruitment^14^. These findings highlight the capacity of PGP to stimulate signaling within non-immune cells, and suggests PGP may alter vascular physiology^15^.

Endothelin-1 (ET1) is a 21-amino-acid peptide produced by endothelial cells^16^ that has multiple pro-inflammatory roles in the heart, lung and kidney^17, 18^. The precursor, big ET1, is activated by endothelin converting enzymes (ECE), thereby liberating ET1 into the circulation and surrounding tissues. ET1 alters ECM regulation^19^, and coronary arterial ET1 expression has been linked with acute rejection following cardiac transplantation^20^. Despite these observations, no study to date has coupled matrikine generation with vascular ET1 expression and disease outcome. Such a mechanism would demonstrate a novel inflammatory paradigm operative in chronic disease. Herein, we show that PGP peptides stimulate ET1 expression, and are likely novel mediators of cardiac rejection after transplantation.

## Results

### Acetylated PGP increases aortic endothelial cell production of ET1 via CXCR2

**Figure 1:**
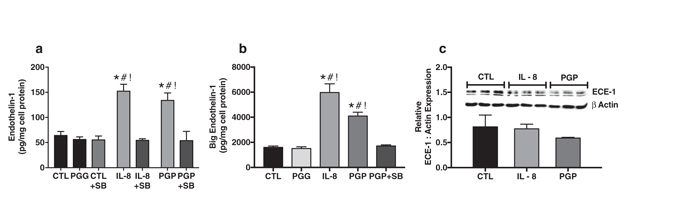
Acetylated PGP increases human aortic endothelial cell production of endothelin-1 via CXCR2 *in vitro*. (**a**) Cultured human aortic endothelial cells were treated with PGG, IL-8, and PGP. Compared to cell lysates from untreated cells (CTL) and PGG, both IL-8 and PGP caused a significant increase in endothelin-1 production (*^#^ *P* < 0.01 compared to CTL and PGG, *n* = 5 experiments). Importantly, pre-treatment with the CXCR2 antagonist SB225002 (SB) ablated the effects of both IL-8 and PGP (^!^ *P* < 0.01 compared to CTL, PGG and SB225002 treated groups, *F* = 14.3). (**b**) Separate experiments further observed that IL-8 and PGP treatment increased big endothelin-1 production from endothelial cell lysates in a CXCR2 dependent manner (*^#!^ *P* < 0.0001 compared to CTL, PGG and PGP co-treated with SB225002, *F* = 32.3, *n* = 3 experiments). (**c**) Neither IL-8 nor PGP altered total protein expression of Endothelin Converting Enzyme (ECE) -1 when assessed by western blot analysis (bar graph from *n* = 3 experiments, *P* = 0.20, *F* = 2.0, inset Illustrates representative immunoblot of ECE-1 compared to β actin control).

Primary human aortic endothelial cells (HAEC) were used for *in vitro* studies, as we have previously shown these cells express CXCR2^13^. HAECs were grown to confluence and treated with interleukin (IL)-8, PGP or the inert negative control peptide Proline-Glycine-Glycine (PGG, see Methods). Compared to untreated control cells, treatment with either IL-8 or PGP increased ET1 production 3-fold (Fig. 1a). PGG had no effect. Furthermore, pre-treatment with the selective CXCR2 antagonist SB225002^21^ ablated IL-8 and PGP dependent changes, demonstrating that activation of CXCR2 is required for stimulation of ET1 production by these ligands (Fig. 1a).

### Acetylated PGP increases aortic endothelial cell production of big ET1 via CXCR2

Endothelial production of ET1 is regulated by cellular transcription and, to a lesser extent, by the expression of ECE^17^. Given these established regulatory mechanisms, we further investigated the effect of PGP on production of the ET1 precursor big ET1. As illustrated in Fig. 1b, *in vitro* administration of PGP to primary HAECs induced a similar 3-fold increase of big ET1. The effect of PGP was similar to IL-8, while administration of PGG had no effect. Co-administration of SB225002 with PGP eliminated big ET1 induction. Finally, neither IL-8 nor PGP altered total protein expression of ECE-1 as assessed by western blot analysis (Fig. 1c). Together, these results clearly demonstrate that PGP induces ET1 production via increased mRNA and protein synthesis as opposed to ECE activation.

### Acetylated PGP increases vascular ET-1 production via CXCR2 *in vivo*

**Figure 2:**
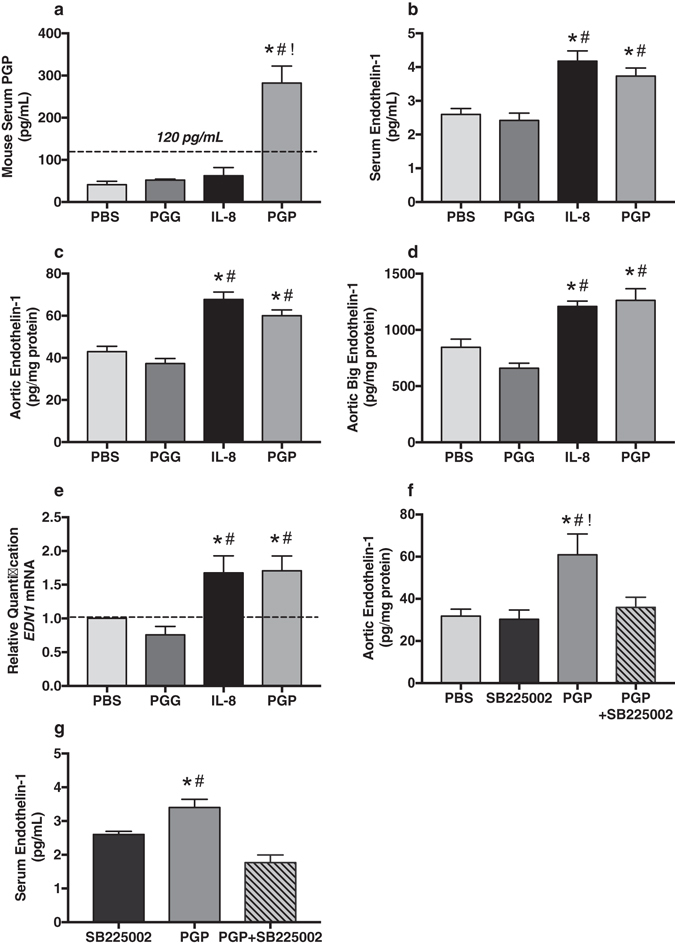
Acetylated PGP increases vascular ET-1 production via CXCR2 *in vivo*. (**a**) As proof of concept, serum concentrations for PGP were measured from c57bl/6 mice systemically treated with PBS, PGG, IL-8 or PGP. Importantly, intraperitoneal administration of PGP increased circulating serum PGP to concentrations comparable to patient samples (*^#!^ *P* < 0.0001, *F* = 23.2, *n* = 4 animals per experimental group). (**b**) Intraperitoneal administration of IL-8 or PGP to c57bl/6 mice significantly increased serum ET1 when compared to PBS and PGG treated mice (*^#^ *P* < 0.0001 compared to PBS and PGG, *F* = 13.07, *n* = 8 animals per group). (**c**) Direct measurement of ET1 in aortic tissue lysates revealed similar results with significant increases in tissue specific ET1 (*^#^ *P* < 0.0001, *F* = 24.7, n = 8 animals per group) and (**d**) big ET1 production (*^#^ *P* < 0.0001*, F* = 18.5, *n* = 8 animals per group). (**e**) Intraperitoneal administration of both IL-8 and PGP significantly increased aortic transcription of Endothelin 1 gene (*EDN1*) mRNA *in vivo* (*^#^ *P* = 0.03 compared to PBS and PGG, *F* = 5.2, *n* = 5 animals per group). Co-administration of PGP and SB225002 (1 mg/kg) blocked the effects of PGP alone on (**f**) aortic ET1 production (*^#!^ *P* = 0.01 compared to PBS, SB225002, and PGP + SB225002, *F* = 5.7, *n* = 8 animals per group) and (**g**) serum ET1 concentrations (*P = 0.02 compared to SB225002 and ^#^ *P* < 0.001 compared to PGP + SB225002, *F* = 5.7, *n* = 5 animals per group). These results demonstrate the importance of CXCR2 activation in ET1 production *in vivo*.

**Figure 3:**
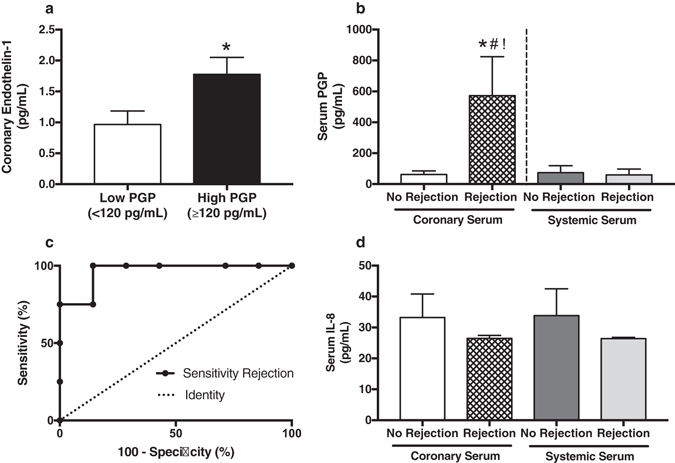
Circulating PGP correlates with vascular endothelin-1 expression and acute cardiac rejection in heart transplant patients. (**a**) In heart transplant patients, coronary serum PGP levels were elevated and associated with coronary ET1 (*P = 0.05 compared to Low PGP, *n* = 11 patients). (**b**) Coronary serum PGP was associated with biopsy proven acute cardiac rejection (*P < 0.01 compared to coronary serum PGP from patients without rejection, *n* = 4 patients with and 7 patients without rejection), and was elevated compared to systemic serum (^#!^ *P* < 0.01). (**c**) ROC curve analysis demonstrated that a cutoff of ≥120 pg/mL of coronary serum PGP corresponded to a sensitivity of 100%, specificity of 86% and positive likelihood ratio of 7 for acute cardiac rejection. The area under the curve (AUC) was 0.96. (**d**) Coronary and systemic serum IL-8 concentrations were measured from patients with and without biopsy proven cardiac rejection (*P* = 0.8, *F* = 0.3, *n* = 4 patients with and 7 patients without rejection). In contrast to PGP, serum IL-8 concentrations were low, unchanged and not associated with acute cardiac rejection. All IL-8 samples were below levels of accurate quantification, but above levels of detection.

To translate our cellular findings to the *in vivo* condition, we intraperitoneally administered PGP, IL-8, PGG or phosphate buffered saline (PBS) to 10-week-old c57bl/6 mice daily over a 2-week period (see Methods). As a proof of concept, mouse serum PGP concentrations were measured from all experimental groups. Intraperitoneally administered PGP significantly increased mouse serum PGP, while PBS, PGG and IL-8 had no effect (Fig. 2a). These mouse serum PGP concentrations were comparable to serum concentrations observed among diseased human subjects^22^ (Figs 2a and 3). *In vivo*, both PGP and IL-8 increased serum ET1 concentrations compared to PBS vehicle control or PGG (Fig. 2b). Direct measurement of ET1 in whole aortic lysates revealed significant ET1 and big ET1 induction in response to systemic PGP or IL-8 administration (Fig. 2c and d). Administration of either IL-8 or PGP significantly increased aortic transcription of ET1 mRNA (*EDN1*) compared to controls (Fig. 2e). In separate experiments, SB225002 (1 mg/kg/dose), PGP (250 µg/dose), or both were intraperitoneally administered daily for two weeks. Compared to controls, administration of PGP similarly increased both aortic (Fig. 2f **)** and serum ET1 (Fig. 2g) concentrations. Importantly, co-administration of SB225002 with PGP blocked these effects, demonstrating the importance of CXCR2 activation in ET1 production *in vivo* (Fig. 2f and g).

### Circulating PGP associates with endothelin-1 expression and acute cardiac rejection

We compared coronary serum PGP and ET1 concentrations in serum from coronary arterial blood obtained from orthotopic heart transplant patients during surveillance left heart catheterization (see Table 1). Coronary PGP levels were elevated (defined as ≥120 pg/mL, t﻿wo standard deviations above the mean coronary serum PGP concentration from patients with no evidence of ﻿rejection) and associated with coronary ET1 (Fig. 3a), while serum IL-8 concentrations were low (means <35 pg/mL) and did not differ between groups (Fig. 3d). Compared with simultaneously collected myocardial biopsies, elevated coronary PGP, but not systemic PGP, associated with cardiac rejection (Fig. 3b, sensitivity 100% and specificity 86%). Receiver operating characteristic (ROC) analysis revealed that a coronary serum PGP concentration of ≥120 pg/mL corresponded to a positive likelihood ratio of 7 for cardiac rejection (Fig. 3c). Finally, we compared coronary serum MMP-9 expression with coronary PGP and rejection status. Elevated coronary PGP demonstrated a trend toward increased active MMP-9 (*P* = 0.09), while cardiac rejection trended toward increased total MMP-9 (*P* = 0.07, Supplementary Figure 1).

**Table 1:** Patient Demographics^*^.

Clinical Group	Age (Years)	Male (%)	Immunosuppressive Medications	History of Smoking (%)
Rejection *(ISHLT* ^†^ *Grade 1 or 2, n* = *4)*	50 ± 2	3 (75.0)	Calcineurin Inhibitors (100%)	2 (50.0)
			Cell-Cycle Inhibitors (100%)	
			Systemic Steroids (100%)	
No Rejection (*n* = *7)*	47 ± 7	6 (85.7)	Calcineurin Inhibitors (100%)	3 (42.9)
			Cell-Cycle Inhibitors (100%)	
			Systemic Steroids (100%)	
*P* value *(between groups)*	ns	ns	ns	ns

^*^Data expressed as mean ± SD or n (%). ^†^ISHLT = International Society for Heart and Lung Transplantation.

## Discussion

The ECM has increasingly been accepted as a dynamic regulator of inflammation. However, the contribution of matrikines to vascular-associated diseases remains poorly understood. While, previous studies have demonstrated that IL-8 can stimulate ET1 production *in vitro* ^23^, no mechanism has been established. Accordingly, this investigation was designed to examine the effects of PGP, a pro-inflammatory matrikine, on endothelial function. Novel findings of this study suggest: (1) PGP is a potent inducer of endothelial ET1 both *in vitro* and *in vivo*; (2) Endothelial CXCR2 activation mediates ET1 production via *EDN1* gene transcription; and (3) Paracrine PGP production is associated with acute myocardial damage and cellular inflammation in acute cardiac rejection following transplantation. Together, these findings identify PGP as the first matrikine capable of inducing endothelial dysfunction and vascular inflammation in association with vascular disease and myocardial injury.

Recent investigations have proposed a role for bioactive collagen fragments following myocardial infarction and remodeling^24^. However, the degree to which ECM fragmentation propagates myocardial and coronary vascular inflammation following transplantation has not been examined. Results from this investigation link previously reported vascular ET1 expression patterns during acute rejection^20^ with a novel pro-inflammatory ECM fragment. Importantly, systemic serum IL-8 concentrations were low and did not differ despite rejection status suggesting a local inflammatory process potentially driven by ECM fragmentation as opposed to canonical cytokine signaling (Fig. 3). Furthermore, experimental results observed trends toward increased MMP-9 coronary serum expression (Supplementary Figure 1), suggesting amplification of established enzymatic cascades for the generation of PGP. Thus, PGP and established mediators may represent a new target in the surveillance and possible treatment of cardiac allograft rejection.

The current study has some notable limitations. Our patient cohort is small, and therefore susceptible to both over and underestimation of the effect size of PGP in cardiac transplant rejection. Likewise, our current study design with sampling of local coronary serum limits the use of a practical validation cohort. Larger clinical studies with validation cohorts are needed before these findings can be reasonably translated to clinical medicine. However, the current results remain promising as a stronger effect may exist for transplant patients presenting with more severe forms of rejection or chronic graft injury.

Independent from cardiac allograft rejection, ET1 is a well-established vasoconstrictive, pro-inflammatory and proliferative agent implicated in vascular dysfunction and cardiovascular disease^25^. Our observation of PGP-induced activation of endothelial CXCR2 and ET1 represents a potential paradigm shift in our understanding of cellular mechanisms responding to vascular injury. More importantly, results from the current investigation underscore a possible role for PGP-induced endothelial dysfunction in other conditions associated with vascular inflammation. Associated pathologies could include stroke^6^, atherosclerosis^26^ and renal disease^27^. Accordingly, future investigations are needed to further elucidate the paracrine role of PGP in other cardiovascular disorders, as well as the relative roles of endothelial CXCR1 and CXCR2 in promoting cytokine-dependent endothelial dysfunction. Our findings emphasize PGP as an underappreciated, non-canonical inflammatory pathway that may be operative in numerous diseases associated with ECM dysregulation.

## Methods

### Reagents

Primary human aortic endothelial cells (HAECs) and vascular cell basal medium supplemented with VEGF (5 ng/mL), EGF (5 ng/mL), FGF (5 ng/mL), IGF-1 (15 ng/mL), L-glutamine (10 mM), heparin sulfate (0.75 U/mL), hydrocortisone (1 µg/mL), ascorbic acid (50 µg/mL), fetal bovine serum (FBS), penicillin (10,000 U/mL), streptomycin (10 mg/mL) and amphotericin B (25 µg/mL) were all obtained from American Type Culture Collection (ATCC, Manassas, VA). Acetylated PGP and PGG were purchased from Bachem (Torrance, CA) and purified to neutrophil chemotactic activity. Human IL-8 with carrier was obtained from Cell Signaling Technology (Danvers, MA). Finally, the selective CXCR2 antagonist SB225002 was purchased from Sigma Aldrich (St. Louis, MO).

### Human Samples

#### Patient demographics

Orthotopic heart transplant patients (*n* = 11 patients) were recruited from the University of Alabama at Birmingham (UAB) Hospital. Patient demographics showed no significant differences in age, sex, smoking history or immunosuppressive medications (Table 1).

#### Myocardial and blood sample collection

Within the first year following transplantation, all patients underwent right heart catheterization with myocardial biopsy and left heart catheterization with coronary angiography as standard of care for cardiac rejection surveillance. Right ventricular biopsies were collected during the same procedure and processed according to UAB Hospital protocols. Independent pathologists reviewed specimens for evidence of rejection according to established International Society for Heart & Lung Transplantation (ISHLT) guidelines^28^. Femoral artery (*systemic*) and Left main coronary artery (*coronary*) blood samples were collected and processed for serum. Specifically, serum samples were stored at room temperature for 30 min in plain tubes and then centrifuged at 10,000 g for 15 min. Serum was aspirated and re-centrifuged at 10,000 g for 15 min to ensure removal of all red blood cells and/or fibrin deposits. Finally, the serum supernatant was removed and stored at −80 °C.

#### Electrospray ionization-liquid chromatography-tandem mass spectrometry

Patient serum samples were filtered through a Millipore (Billerica, MA) 10,000 molecular weight cutoff centrifugal filter followed by washing with 30 µL of 1 mM HCl. Acetylated PGP was measured using a MDS Sciex API-4000 spectrometer (Applied Biosystems, Carlsbad, CA) equipped with a Shimadzu HPLC (Columbia, MD). HPLC was performed using a 2.0_150-mm Jupiter 4 u Proteo column (Phenomenex, Torrance, CA) with A: 0.1% HCOOH and B: MeCN + 0.1% HCOOH: 0–0.5 min 5% buffer B/95% buffer A, then increased over 0.5–2.5 min to 100% buffer B/0% buffer A. Background was removed by flushing with 100% isopropanol/0.1% formic acid. Positive electrospray mass transitions were at 312–140 and 312–112 of acetylaed PGP.

### Animal Model

#### Murine studies

Healthy 8- to 10-week-old c57bl/6 male mice (The Jackson Laboratory, Bar Harbor, ME) were used in these studies. Phosphate buffered saline (PBS, 250 µL) containing 250 µg of PGP was intraperitoneally administered daily for two weeks. PBS and PBS containing 250 µg PGG or 100 ng IL-8 served as controls. In separate experiments, SB225002 (1 mg/kg/dose), PGP (250 µg/dose), or both were intraperitoneally administered daily for two weeks. SB225002 was dissolved in PBS with 0.3% Tween-20 to a final volume of 210–250 µL per dose. Twenty-four hours after the last dose, mice were anesthetized by intraperitoneal injection of ketamine (120 mg/kg) and xylazine (8 mg/kg). Through a 1-cm abdominal midline incision, blood was collected by intracardiac puncture before euthanasia. The thoracic aorta was isolated, rinsed in PBS and dissected clear of surrounding adipose and connective tissues. Whole aortas were frozen in liquid nitrogen and stored at -80 °C before processing for total protein and/or RNA isolation.

#### Murine tissue preparation

Murine blood samples were centrifuged at 3500 rpm for 15 min, and serum was aspirated and stored at −80 °C. Murine aortic tissue assessed for enzyme-linked immunosorbent assays (ELISA) was removed immediately after sacrifice and snap-frozen in liquid nitrogen. The tissue was lysed with RIPA lysis buffer (Santa Cruz Biotechnology, Dallas, TX) and homogenized. Lysates were clarified by centrifugation at 14,000 g for 10 min, and protein concentration as determined by BCA assay (Bio-Rad, Hercules, CA). Total RNA from murine aortic tissue assessed for real-time PCR was isolated using Aurum total RNA mini kit reagents and protocol (Bio-Rad).

### *In vitro* experiments

#### Cell culture

Primary HAECs were maintained as previously described^13^ and all experiments were performed on passage four or less cells. Cells were grown to confluence and serum starved one hour before a four-hour stimulation with PGP (1 mg/mL), PGG (1 mg/mL), or IL-8 (100 ng/mL). Where indicated, cells were pretreated for one-hour with SB225002 (200 nM) prior to experimentation. Following stimulation, cell lysates were processed with RIPA lysis buffer, measured for protein concentrations and stored at −80 °C.

#### ELISA

ET1, IL-8, total and active MMP-9 concentrations for serum samples, aortic endothelial cell lysates and aortic tissue protein lysates were measured using commercially available ELISA kits and protocols from R&D Systems (Minneapolis, MN). Results from cell and tissue lysates were normalized to sample total protein concentrations. Big ET1 concentrations were similarly measured with human big ET1 ELISA kit from My Bio Source (San Diego, CA).

#### RT-PCR and TaqMan gene expression assays

Total RNA was isolated from murine aortic lysates (see *Murine tissue preparation*), and RT reactions were performed as previously described^29^. Five hundred nanograms of RNA was used to reverse transcribe into cDNA and subjected to qRT-PCR. The data were analyzed using the comparative Ct method to obtain relative quantitation values.

#### Western blot

Samples were resolved using electrophoresis on a 15% polyacrylamide gel followed by transfer to a 0.2 µm nitrocellulose membrane. Blots were blocked with 5% bovine serum albumin in TBS + 0.1% Tween 20 (TBST) and incubated overnight at 4 °C with antibodies against ECE-1 (Abgent, San Diego, CA) and β actin (Sigma Aldrich). Blots were washed in TBST, incubated with species-appropriate HRP-conjugated secondary antibody and washed again in TBST. Signals were detected using enhanced chemiluminescence and x-ray film. ImageJ software was used to quantify immunoflorescent bands.

### Statistics

All results are displayed as means ± SEM. For multiple comparisons, ordinary one-way ANOVA and post hoc analysis (Tukey) tests were applied. The Student’s *t* test was used for comparisons of the mean values of two different samples. *P* ≤ 0.05 was considered significant. Receiver operating characteristic analysis was used to determine the optimal PGP concentration associated with cardiac rejection. All data was normally distributed. All statistical analyses were performed using GraphPad Prism software (La Jolla, CA).

### Study Approval

Human and animal studies were approved by the appropriate institutional review boards. Specifically, all animal studies were approved by the UAB Institutional Animal Care and Use Committee (protocol number 10075). Regarding human studies, approval was granted by the UAB Institutional Review Board (IRB 00000726). Written informed consent was received from all participants prior to inclusion in the study. All human and animal studies were performed in accordance with relevant guidelines and regulations.

### Data Availability

The datasets generated during and/or analyzed during the current study are available from the corresponding authors on reasonable request.

## Electronic supplementary material

Supplementary Information
