# Supplementary material for: The Matrikine Acetylated Proline-Glycine-Proline Couples Vascular Inflammation and Acute Cardiac Rejection
Source: Sci Rep. 2017 Aug 8;7:7563. doi: 10.1038/s41598-017-07610-0 (PMC5548740; doi:10.1038/s41598-017-07610-0)
Supplement: Supplementary file 1 — Supplementary Information [file 41598_2017_7610_MOESM1_ESM.pdf]

Supplementary Information:

The Matrikine Acetylated Proline-Glycine-Proline Couples Vascular Inflammation and Acute Cardiac Rejection

Gregory A. Payne MD, PhD[1,3,4*], Jindong Li[2,4], Xin Xu MD, PhD[2,4], Patricia Jackson PhD[2,4-6], Hongwei Qin PhD[7], David M. Pollock PhD[9], J. Michael Wells MD[2,4-6], Suzanne Oparil MD[1,3,7], Massoud Leesar MD[1,6], Rakesh P. Patel PhD[4,8], J. Edwin Blalock PhD[2,4,5,7†] and Amit Gaggar MD, PhD[2-7*†]

[1]*Division of Cardiovascular Disease, Department of Medicine, University of Alabama at Birmingham, Birmingham, AL*
[2]*Division of Pulmonary, Allergy & Critical Care Medicine, Department of Medicine, University of Alabama at Birmingham, Birmingham, AL*
[3]*Vascular Biology and Hypertension Program, University of Alabama at Birmingham, Birmingham, AL*
[4]*Program in Protease and Matrix Biology, University of Alabama at Birmingham, Birmingham, AL*
[5]*Lung Health Center, University of Alabama at Birmingham, Birmingham, AL*
[6]*Medical Service at Birmingham VA Medical Center, Birmingham, AL*
[7]*Department of Cell, Developmental, and Integrative Biology, University of Alabama at Birmingham, Birmingham, AL*
[8]*Department of Pathology and Center for Free Radical Biology, University of Alabama at Birmingham, Birmingham, AL*
[9]*Cardio-Renal Physiology and Medicine, Division of Nephrology, Department of Medicine, University of Alabama at Birmingham, Birmingham, AL*
**denotes corresponding authors*
[†]*denotes equal contribution*

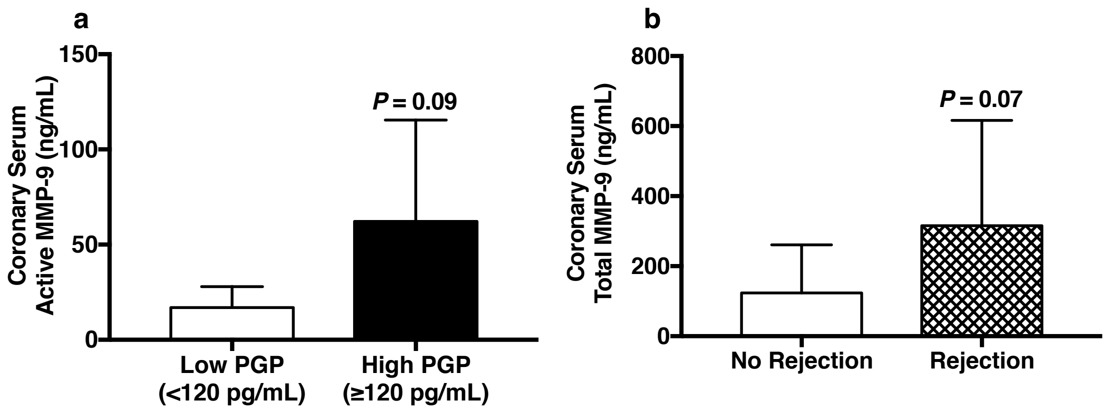

Supplementary Figure 1. **MMP-9 expression associates with PGP generation and acute cardiac rejection.** (a) In heart transplant patients, coronary serum PGP levels were elevated and associated with a trend toward increased active MMP-9 within coronary serum (*P* = 0.09 compared to Low PGP, *n* = 11 patients). (b) Similarly, patients with biopsy proven acute transplant rejection trended toward increased coronary serum total MMP-9 (*P* = 0.07 compared to No Rejection, *n* = 11 patients).
